# GEOGRAPHIC DISTRIBUTION OF SENIOR SUBSIDIZED HOUSING IN NEW JERSEY: GEOGRAPHIC INFORMATION SYSTEMS APPROACH

**DOI:** 10.1093/geroni/igae098.1960

**Published:** 2024-12-31

**Authors:** Takashi Amano, Sojung Park, Byeongju Ryu, Jihye Baek

**Affiliations:** Rutgers, The State University of New Jersey, Newark, New Jersey, United States; Washington University in St. Louis, St. Louis, Missouri, United States; Washington University in St. Louis, St. Louis, Missouri, United States; Washington University in St. Louis, St. Louis, Missouri, United States

## Abstract

Subsidized senior housing (SSH) offers a solution for low-income older individuals, enabling them to maintain economic stability and continue living independently. There has been limited investigation into the geographical distribution of SSH and its association with neighborhood disadvantage. This study seeks to address this gap by (1) describing the availability of SSH in New Jersey and (2) exploring the extent to which housing availability is linked to neighborhood disadvantage. SSH-availability was quantified by the number of SSH units within each census block group. Neighborhood disadvantage was measured using the area deprivation index (ADI). The eligible population was defined as residents aged 62 or older, living below the poverty line, and allocating 30% or more of their income to housing costs within the census block group. A Geographic Information System (GIS) was employed to visually analyze the spatial distribution of SSH, ADI scores, and eligible populations across New Jersey. Poisson regression was utilized to assess the relationship between SSH availability and neighborhood disadvantage, adjusting for the number of eligible residents. The results revealed a significant association between higher SSH availability and higher ADI scores after accounting for the number of eligible residents (p<.001). This suggests that SSH tend to be located in areas experiencing greater economic deprivation. These findings underscore the need for governmental intervention to mitigate geographic disparities in SSH provision for low-income older individuals. Additionally, further investigation is warranted to examine the accessibility of social and health-related resources surrounding SSH, which could support residents aging in place.

